# An Assessment of Forage Selection by Giraffe Introduced into Umfurudzi Park, Northern Zimbabwe

**DOI:** 10.1155/2018/9062868

**Published:** 2018-07-24

**Authors:** Takunda V. Munyaka, Edson Gandiwa

**Affiliations:** School of Wildlife, Ecology and Conservation, Chinhoyi University of Technology, Private Bag 7724, Chinhoyi, Zimbabwe

## Abstract

Giraffe (*Giraffa camelopardalis*) is one of the flagship herbivore species in the savanna ecosystem and is of high conservation value. Management of the species under diversified ecosystems, particularly, their introduction in new ecosystems is of great concern, given that limited information is available of how the species acclimatizes to new ecosystems and which forage species it selects. The objectives of the present study were to (i) identify woody plant species selected by the recently introduced giraffes and (ii) determine whether there were differences in woody plant diversity between the dry and wet seasons in Umfurudzi Park, northern Zimbabwe. Forage selection and woody composition data were collected from a herd of giraffe between May and December 2016, using the focal observation method in an enclosure within the study area. A total of 106 observation plots were established. Our results showed that 12 woody plant species comprising six families were selected from a total of 29 woody plant species recorded in the study area. Giraffe showed a higher preference of the selected species in the dry season than in the wet season. In contrast, no significant differences were recorded in terms of forage availability and woody vegetation diversity between seasons. In conclusion, our results suggest that plant phenology, particularly, presence of leaves on plants influences giraffe feed preferences. Establishing long-term monitoring plots to determine woody vegetation utilisation by giraffes is valuable as a way to monitoring habitat utilisation by the species.

## 1. Introduction

Giraffes (*Giraffa camelopardalis*) are browsing ruminants in the family Bovidae [1]. According to Gordon et al. [1], giraffes mainly browse on woody plant parts and forbs and feed selectively. The translocation of giraffes has been done for a number of reasons including the process of rehabilitation, and thus, the successes of giraffe translocation have shown that giraffes can adapt to different ecosystems [2].

Several studies examined the feeding behavior of giraffes in Africa [3–5]. These studies have reported forage selectivity by giraffes [4–6] across seasons [3]. Intake of forage by giraffes is strongly affected by the plant defense mechanisms [7]. The likelihood of a plant species being eaten when encountered by an herbivore can be defined as the acceptability of that plant species to the herbivore [8]. Furthermore, Arnold [9] defined availability as relating to frequency of occurrence, relative yield, and accessibility, as well as the nature of the surrounding plant material and that this will influence acceptability to animals.

Most wild animals that are introduced into new areas have to go through a process of acclimatization and adaptation [2]. Of interest is that giraffes have proved to adapt easily to new environments and do not significantly affect other species that may be present in the areas they are introduced [2]. A recent introduction of giraffes was recorded in Umfurudzi Park, northern Zimbabwe. However, there is limited information of how the giraffes are utilising the habitat in terms of species preferred between the dry and wet season. Therefore, the objectives of the present study were to (i) identify woody plant species selected by the recently introduced giraffes and (ii) determine whether there were differences in woody plant diversity between the dry and wet seasons in Umfurudzi Park. The study results will provide a baseline for future assessment of plant species utilisation patterns by giraffes and habitat changes.

## 2. Materials and Methods

### 2.1. Study Area

Umfurudzi Park, northern Zimbabwe, lies between latitudes 17° 15′ and 16° 50′ south and 31° 40′ and 32° 00′ north with altitude that ranges from 740 to 1020 m. The park covers an area of 760 km^2^ and is located approximately 150 km from Harare [10]. The park has a mean annual rainfall of 550 mm [10]. Umfurudzi Park is divided into two seasons, namely, the dry and wet seasons. The wet season starts in November and ends in April, whereas the dry season begins in May and ends in October. The park is located in a Miombo ecoregion, an area dominated by the following plant species: *Brachystegia boehmii*, *Colophospermum mopane*, and *Julbernardia globiflora* [11]. As part of the park rehabilitation programme, approximately 1,800 animals including 43 giraffes (25 in 2012 and 18 in 2013) were either reintroduced or introduced into Umfurudzi Park between 2010 and 2013. The studied giraffes were confined to an enclosure with an area of about 30 km^2^. The enclosure is part of the Umfurudzi Park fenced to allow for animal acclimatization before the fence is removed to allow animals to utilise the wider park habitats.

### 2.2. Data Collection

A systematic random sampling technique was used in selecting a focal animal to observe from the identified herd of giraffes, which consisted of eight individuals. Walked transects were used to traverse the enclosure as these minimise disturbance to animals [12]. Data collection was conducted during the day between 06:30 and 18:30 hours, for the period 1st of May 2016 to 30th of December 2016. Field data collection activities were divided into two, that is, focal observation and vegetation assessment. When any one giraffe from the study herd was observed browsing, the woody plant browsed was noted from a distance using binoculars following methods outlined by Muposhi et al. [10]. The woody plant was visited after the giraffe had moved to other plants so as not to influence the giraffe-browsing patterns. A circular plot with a radius of 15 m was established around the browsed plant so as to assess the diversity of woody plants at each focal point. Overall, a total of 106 circular observation plots (71 for dry season and 35 for wet season) were established and assessed. The plant species names were recorded with the aid of tree identification guides.

### 2.3. Data Analysis

Mapping of the woody plant species utilisation by giraffes over the study period was conducted, and woody species diversity was determined using the Shannon–Weiner Index (*H*′) [13]. Giraffe preference was quantified through determining the acceptability and availability indices [14]. The availability index was calculated by dividing the number of observation points in which the woody plant species was present by the total number of observation plots, whereas acceptability was calculated by dividing the number of observation plots in which the woody species was foraged by the number of observation plots in which the species was present.

Data related to seasonal availability and acceptability and woody vegetation composition were tested for normality using the Shapiro–Wilk test and found to be not normal. Thus, the Mann–Whitney *U* test was used to determine whether there were significant differences for the study variables between the dry and wet seasons using the Statistical Package for Social Sciences (SPSS) version 16.0 for Windows (SPSS Inc., Chicago).

## 3. Results

A total of 29 woody plant species were recorded from the 106 observation plots with 12 woody plant species being selected by the introduced giraffe. *Piliostigma thonningii*, *Vangueria infausta*, *Ziziphus mucronata*, *Combretum apiculatum*, *Pseudolachnostylis maprouneifolia*, and *Combretum molle* were preferred during the dry season, whereas *Flueggea virosa*, *Olea africana*, *Combretum erythrophyllum*, *Combretum hereroense*, *Combretum imberbe*, and *Ziziphus mucronata* were preferred in the wet season. These species represented five families. *A. karroo* was also recorded as forage for both dry and wet seasons. Giraffes selected more than three species in each month during the study period (Table 1).

Overall, there was a moderate availability of woody plants browsed by the giraffes throughout the dry and wet seasons. Accordingly, there was a no significant difference in the availability of woody species browsed by giraffes between the dry and wet seasons in Umfurudzi Park (*P* > 0.05; Table 2). A significant difference in acceptability was recorded (*P* < 0.05; Table 2) with a total of 10 and 7 woody plant species being selected by giraffes in Umfurudzi Park in the dry and wet seasons, respectively (Table 3). There was a no significant difference in woody species diversity between the dry and wet season in Umfurudzi Park (Mann–Whitney *U* test, *P* > 0.05). Woody plant species from the Combretaceae family, that is, *C. imberbe*, *C. hereroense*, and *C. erythrophyllum* and those from Rhamnaceae family (i.e., *Z. mucronata*) were highly acceptable in the dry season. In the wet season, *C. apiculatum* from the Combretaceae family was the mostly acceptable species (Table 3).

## 4. Discussion

We recorded 12 tree species from six families (i.e., Combretaceae, Fabaceae, Phyllanthaceae, Oleaceae, Rubiaceae, and Rhamnaceae) that were preferably browsed by giraffes in Umfurudzi Park in the dry and wet seasons. Our results showed that most plant species selected by giraffes were from the family Combretaceae. Most of the recorded preferred woody plant species were frequently browsed in both dry and wet seasons. An earlier study reported that in the southern parts of Zimbabwe, *Acacia* was recorded as the most frequently browsed woody plant genus [4]. In this present study, there was evidence of *Acacia* being selected throughout both seasons showing that the genus is preferred also in Miombo woodlands. However, it was evident that giraffes also expanded their forage selection as evidenced by the 12 recorded and selected species; hence, this infers their possible acclimatization in the Miombo ecosystem.

Our results showed a variation in tree species selected by giraffes between the dry and wet seasons. It has been suggested that herbivores select diets based on the concentration of nutrients relative to toxins [7]. Giraffes are known to select woody plant species on the basis of their nutritive value as well as the greenness (e.g., *C. erthrophylum*) [15]. For instance, *C. erythrophyllum* was preferred during the dry season more than in the wet season in the present study.

Previous studies on giraffe diet have indicated that deciduous species such as *A. karroo* dominate the vegetation of habitats used by giraffes and make up the bulk of the diet during the wet season [16]. The present study differs from these earlier studies since the study area is predominantly a Miombo ecosystem; hence, providing a broad range of other species compared to the mopane-dominated savanna ecosystem. During the dry season, however, these deciduous plants lose their leaves and the giraffes tend to concentrate along riverine habitats where they feed on the evergreen vegetation [16].

A significant seasonal variation in the acceptance of the woody plants browsed by giraffes was recorded in this present study. Differences in the acceptance of tree species by the giraffe in relation to seasonality can be attributed to woody plant species selectivity by giraffes between the dry and wet seasons [17]. It has been reported that high acceptance is influenced by favorable nutritive values of the forage species [5] among other factors. The fact that some of the tree species were selected in both dry and wet seasons suggests that woody plant availability had a little influence on forage selection of giraffes. Omphile et al. [18], however, reported that seasonal selection of forage by herbivores can be influenced by plant availability. It has been reported that herbivore species will only switch diet if there are issues to do with nutritive value or digestibility of the feed [14]. However, from the present study, forage availability was almost the same in both the dry and wet seasons as evidenced by the nonsignificant difference in woody species plant diversity.

From field observations in this present study, it was evident that during the dry season, giraffes concentrated in the riparian zone and browsed on species such as *C. erythrophyllum* and *C. molle*. During the wet season, giraffes switched to plant species such as *F. virosa*. Thus, there was evidence to show that giraffes selected the most nutritious and available tree species within each season [5]. Change in seasons mean that the trees also go through variations such as leaf shading, fruits ripening, and in some cases death of plants [5]. These variations affect the forage selection by giraffes. For instance, field observations showed that *A. karroo* had fruits during the dry season and leaves during the wet season. Thus, giraffes could utilise leaves in the wet season and the fruits during the dry season.

Given that wild animals mostly search for plant species that provide them with the best nutrients, it is thus likely that the differences in selected plant species is influenced by choices for nutrients and also plant defense systems, for example, thorns and tannins. Therefore, it is likely that the plant phenology plays an important role in influencing herbivory patterns by giraffes in the study area.

## 5. Conclusion

The giraffes in Umfurudzi Park selected a wide spectrum of woody plant species, that is, 12 species from the 29 species that were recorded in this present study. The results show a significant variation in the acceptability of woody plant species with giraffes showing high acceptability of certain species in the dry season than in the wet season. Furthermore, the present study showed that there was a no significant difference in woody vegetation composition in the study site in Umfurudzi Park between the dry and wet seasons. It is possible that giraffes were restricted to the few woody plant species found in the enclosure. Thus, it is recommended that future studies examine the parkwide forage selection by the giraffes so as to develop a baseline for future monitoring of habitat use by wild animal species within the park.

## Figures and Tables

**Table 1:** Mapping woody species selected by giraffes during the study period in Umfurudzi Park.

#	Species name	Family	Browse preferences							
			Dry season						Wet season	
			MAY	JUN	JUL	AUG	SEP	OCT	NOV	DEC
1	*Acacia karroo*	Fabaceae	DS	DS	DS	DS			WS	WS
2	*Balanites aegyptiaca*	Zygophyllaceae								
3	*Burkea africana*	Fabaceae								
4	*Brachystegia boehmii*	Fabaceae								
5	*Bauhinia galpinii*	Fabaceae								
6	*Bauhinia petersiana*	Fabaceae								
7	*Brachystegia spiciformis*	Fabaceae								
8	*Combretum apiculatum*	Combretaceae			DS					WS
9	*Combretum erythrophyllum*	Combretaceae					DS	DS	WS	
10	*Croton gratissimus*	Euphorbiaceae								
11	*Combretum hereroense*	Combretaceae	DS			DS	DS	DS		
12	*Combretum imberbe*	Combretaceae					DS		WS	WS
13	*Combretum molle*	Combretaceae		DS						
14	*Colophospermum mopane*	Fabaceae								
15	*Commiphora mossambicensis*	Burseraceae								
16	*Diplorhynchus condylocarpon*	Apocynaceae								
17	*Flueggea virosa*	Phyllanthaceae						DS		WS
18	*Julbernardia globiflora*	Fabaceae								
19	*Lantana camara*	Verbenaceae								
20	*Lannea discolour*	Anacardiaceae								
21	*Olea africana*	Oleaceae						DS	WS	
22	*Pseudolachnostylis maprouneifolia*	Phyllanthaceae				DS				
23	*Piliostigma thonningii*	Fabaceae	DS							
24	*Terminalia brachystemma*	Combretaceae								
25	*Terminalia sericea*	Combretaceae								
26	*Terminalia stenostachya*	Combretaceae								
27	*Vangueria infausta*	Rubiaceae		DS	DS					
28	*Ximenia caffra*	Olacaceae								
29	*Ziziphus mucronata*	Rhamnaceae	DS	DS	DS					

DS = dry season; WS = wet season.

**Table 2:** Forage indices and species diversity (median ± range) of woody plants selected by giraffes in the wet and dry season in Umfurudzi Park.

Variable	Season		Mann–Whitney *U*	*df*	*P* value
	Dry season	Wet season			
Acceptability	0.36 ± 0.75	0.03 ± 1.02	29.00	1	0.014
Availability	0.14 ± 0.50	0.08 ± 0.55	56.50	1	0.386
Woody plant diversity (*H*′)	1.40 ± 1.40	1.45 ± 1.90	1089.50	1	0.183

**Table 3:** Seasonal availability and accessibility of woody plant species browsed by giraffes in Umfurudzi Park.

Species name	Family	Dry season		Wet season	
		Availability	Acceptability	Availability	Acceptability
*A. karroo*	Fabaceae	0.50	0.35	0.42	0.10
*C. hereroense*	Combretaceae	0.19	**0.60**	0.25	0.05
*C. molle*	Combretaceae	0.33	0.23	0.55	0.00
*C. apiculatum*	Combretaceae	0.13	0.36	0.13	**1.00**
*C. imberbe*	Combretaceae	0.08	**0.75**	0.04	0.03
*C. erythrophyllum*	Combretaceae	0.14	**0.53**	0.05	0.04
*F. virosa*	Phyllanthaceae	0.00	0.00	0.10	0.07
*P. thonningii*	Fabaceae	0.08	0.44	0.00	0.00
*P. maprouneifolia*	Phyllanthaceae	0.48	0.19	0.00	0.00
*O. africana*	Oleaceae	0.00	0.00	0.44	0.03
*V. infausta*	Rubiaceae	0.43	0.13	0.00	0.00
*Z. mucronata*	Rhamnaceae	0.11	**0.75**	0.00	0.00

*Note*. Dry season represents May to October and wet season represents November and December. Numbers in bold represent the woody plant species which were highly selected.

## Data Availability

The data used to support the findings of this study are available from the corresponding author upon request.
